# Optimization of an Injectable Hydrogel Depot System for the Controlled Release of Retinal-Targeted Hybrid Nanoparticles

**DOI:** 10.3390/pharmaceutics15010025

**Published:** 2022-12-21

**Authors:** Ilaria Ottonelli, Andrea Bighinati, Elisa Adani, François Loll, Riccardo Caraffi, Maria Angela Vandelli, Frank Boury, Giovanni Tosi, Jason Thomas Duskey, Valeria Marigo, Barbara Ruozi

**Affiliations:** 1 Clinical and Experimental Medicine PhD Program, University of Modena and Reggio Emilia, 41124 Modena, Italy; 2 Nanotech Lab, Te.Far.T.I., Department of Life Sciences, University of Modena and Reggio Emilia, 41125 Modena, Italy; 3 Department of Life Sciences, University of Modena and Reggio Emilia, 41125 Modena, Italy; 4 Inserm UMR 1229 RMeS Regenerative Medicine and Skeleton, CHU Nantes, Univ Angers, Nantes Université, Oniris, 44000 Nantes, France; 5 Inserm UMR 1307, CNRS UMR 6075, Université de Nantes, CRCI2NA, 49000 Angers, France; 6 Center for Neuroscience and Neurotechnology, via Campi 287, 41125 Modena, Italy

**Keywords:** nanomedicine, targeted nanoparticles, injectable hydrogel, hyaluronic acid, poloxamer 407, PLGA, intravitreal injection, retinal targeting

## Abstract

A drawback in the development of treatments that can reach the retina is the presence of barriers in the eye that restrain compounds from reaching the target. Intravitreal injections hold promise for retinal delivery, but the natural defenses in the vitreous can rapidly degrade or eliminate therapeutic molecules. Injectable hydrogel implants, which act as a reservoir, can allow for long-term drug delivery with a single injection into the eye, but still suffer due to the fast clearance of the released drugs when traversing the vitreous and random diffusion that leads to lower pharmaceutic efficacy. A combination with HA-covered nanoparticles, which can be released from the gel and more readily pass through the vitreous to increase the delivery of therapeutic agents to the retina, represents an advanced and elegant way to overcome some of the limitations in eye drug delivery. In this article, we developed hybrid PLGA-Dotap NPs that, due to their hyaluronic acid coating, can improve in vivo distribution throughout the vitreous and delivery to retinal cells. Moreover, a hydrogel implant was developed to act as a depot for the hybrid NPs to better control and slow their release. These results are a first step to improve the treatment of retinal diseases by protecting and transporting the therapeutic treatment across the vitreous and to improve treatment options by creating a depot system for long-term treatments.

## 1. Introduction

The retina, located in the posterior segment of the eye, is part of the Central Nervous System (CNS) and is responsible for light sensitivity, which is why retinal pathologies affecting different cell types in the retina are directly related to sight loss and blindness. Several multifactorial disorders can result in retinal impairments, such as diabetes, retinal detachment, age-related retinal degenerations and tumors, or inherited forms of retinal degeneration. These are an important burden to society because almost 300 million people suffer from severe visual impairment and blindness worldwide [1,2,3]. The retina is well protected by both the blood–retinal barrier and the inner limiting membrane (at the retinal–vitreal interface), regulating the flux into and out of the retina, which greatly limits administration routes. Eye drops are a common drug delivery system for the eye, but this administration route poorly reaches the retina [4]. Surgery is still the major treatment option to remove damaged tissues, administer therapeutic molecules, or place implants and scaffolds for the long-term release of drugs, but it is poorly tolerated by patients despite recent advances in surgical techniques [5,6]. Subretinal injections are very effective in the administration of drugs to the retina, as demonstrated in the delivery of Luxturna^TM^, an approved gene therapy for a severe form of retinal degeneration [7]; however, this treatment requires a single injection and not repetitive administration. One of the most frequently used routes is intravitreal (IVT) injection, which is less invasive and more tolerated compared to other techniques, allowing for repeated injections but increasing the risk of inflammatory reactions in the eye and retinal detachment [8,9]. Non-specific diffusion through the eye can lead to poor accumulation in the retina and, finally, free drugs often suffer short half-lives and poor biodistribution through the vitreous when attempting to deliver them to the retina [10,11].

An approach to overcome these limitations, reducing the frequency of administration and improving specific retinal targeting, could be to load therapeutic molecules into nanoparticles (NPs). NPs have emerged as effective tools to deliver therapeutics to specific cells [12,13,14]. In fact, NPs can be tuned to vary their chemico-physical properties and engineered with specific surface modifications to specifically interact with target cells [15,16,17]. Moreover, NPs have the ability to encapsulate and protect sensitive molecules from degradation in the biological environment, such as peptides, enzymes, and genetic materials, which have been proposed for their therapeutic effects but degrade rapidly in vivo, as is often the case when delivering to the retina [18,19]. All these advantages can help reduce side effects to the surrounding healthy tissues and increase the overall therapeutic efficacy of drugs for retinal pathologies, while decreasing the necessary dose and lowering the number of injections. The size of the NPs can be tuned to have specific behavior after IVT injection: it has been demonstrated that NPs larger than 500 nm tend to be rapidly eliminated from the vitreous, while NPs between 200 and 250 nm are cleared less rapidly, offering beneficial delivery potential [10,20]. Finally, the surface of NPs can be modified to improve their mobility and avoid clearance by immune cells, which is the starting point for an efficient drug delivery [21,22].

Despite their advantageous properties, when in the vitreous, NPs can interact with anionic macromolecules, resulting in their retention at the injection site where they can be eliminated by hyalocytes or freely diffuse and be cleared from the vitreous, hampering their long-term efficacy and still requiring multiple injections [23,24,25,26,27]. Moreover, direct injection of NPs might cause toxicity due to poor biomimetic properties of the medium and poor control on drug release with high burst release [28]. To improve their therapeutic profile, reduce their clearance and, therefore, the needed number of administrations, while maintaining the advantageous properties of NPs, a depot system can be used. Hydrogels are currently on the rise as effective depot drug delivery systems thanks to their properties, such as biocompatibility, biodegradability, high stability, and sensitivity to specific stimuli, that can lead to gelification (pH and temperature) after in vivo administration [29,30]. Several studies have already shown the advantages of hydrogels for ocular delivery to improve the durability and slow release of large molecules that normally do not transfer through the vitreous, such as growth factors, antibiotics, or anti-inflammatory drugs, and as scaffolds for wound healing, tissue regeneration after brain surgery, bone regeneration, or spinal cord injuries [31,32,33,34,35,36,37,38,39]. For this reason, hydrogels have also been loaded with NPs, to promote drug loading and improve drug delivery strategies by exploiting the features of the NPs; however, most of these studies focus on inorganic NPs, such as gold, silica, or silver NPs [40,41,42], that offer high stability and reproducibility but tend to accumulate in the tissues and can cause long-term toxicity [43,44]. Finally, depending on the makeup of the hydrogel, their stability and degradation rate can be tuned from a few days to several weeks, as shown with other similar hydrogels in the literature [45,46,47,48]. This will become critical when deciding on the desired release kinetics for retinal treatment.

In this study, we present an injectable hydrogel loaded with retinal-targeted, biodegradable, hybrid NPs. This system can act as a depot to improve retinal delivery of therapeutics after IVT injection to both protect the therapeutic molecules and slow down their release for more long-term treatment options. Hybrid NPs were formulated with biodegradable components, such as poly(L-lactide-co-glycolic) acid and phospholipids, which were fully characterized for their physical chemical properties. Retinal targeting was achieved through a surface functionalization of the NPs with hyaluronic acid and their endocytosis by retinal photoreceptors was assessed in vitro. To control the release of the NPs, they were embedded into a thermosensitive hydrogel composed of poloxamer 407 and hyaluronic acid, which showed a liquid behavior at 4 °C but underwent gelification at 37 °C. These results demonstrated that the hydrogel could delay the mobility of NPs for up to 36 h after administration. This complex delivery system could greatly improve the administration of sensitive therapeutic molecules to the retina by exploiting both the targeting ability and protective effect of NPs while prolonging their release, improving the potential therapeutic timeframe with injectable hydrogels.

## 2. Materials and Methods

### 2.1. Materials

The Resomer^®^ RG 503H Poly (D,L-lactide-co-glycolide) 50:50 (PLGA) MW 11,000-12,000 was purchased from Evonik (Essen, Germany). Dioleoyl trimethylammonium propane (DOTAP) was purchased by Avanti Polar Lipids (Alabaster, AL, USA). Poloxamer 188; poloxamer 407; Extra-Low-molecular-weight hyaluronic acid MW 8–12 kDa (HA12); acetone; methanol; trifluoroacetic acid (TFA); barium chloride (BaCl_2_); iodine (I_2_); and low-gelation-point agarose were purchased from Sigma Aldrich (St. Louis, MO, USA). Hyaluronic acid, MW 330 kDa (HA330), and 1100 kDa (HA1100) were purchased from Contipro (Dolní Dobrouč, Czech Republic). Cy5-PLGA was purchased from CD-Bioparticles (Shirley, NY, USA). Dichloromethane (DCM) and potassium iodide (KI) were purchased from Carlo Erba (Cornaredo, Italy). MilliQ water was purified by a Millipore system (Millipore, Bedford, MA, USA). All chemicals used were of analytical grade. Avertin mixture (1.25% (*w*/*v*) 2,2,2-tribromoethanol and 2.5% (*v*/*v*) 2-methyl-2-butanol), Davidson’s fixative (8% Formaldehyde, 31.5% Ethanol, 2 M Acetic Acid), Paraffin, Paraformaldehyde, 4’,6-diamidino-2- phenylindole (DAPI), Mowiol 4-88, and 3-(4,5-dimethylthiazol-2-yl)-2,5-diphenyl tetrazolium bromide (MTT) were purchased from Sigma Aldrich. Anti-β-Tubulin and anti-Rhodopsin clone 1D4 were purchased from Sigma Aldrich. Anti-Iba1 was purchased from Wako-Chemicals (Fujifilm-Wako, Neuss, Germany). Secondary antibodies anti-mouse and anti-rabbit AlexaFluor 488 were purchased from Invitrogen (Thermo Fisher Scientific, Waltham, MA, USA). 661W-A11 cells [49] were derived from 661W cells received from Dr. Muayyad Al-Ubaidi [50] and are a photoreceptor cell line. Dulbecco’s Modified Eagle Medium, Trypsin, Fetal Bovine Serum, Glutamine, and Penicillin–Streptomycin were purchased from Thermo Fisher Scientific (Waltham, MA, USA).

### 2.2. Nanoparticles

#### 2.2.1. NP Formulation

NPs were produced following the nanoprecipitation protocol adapted from previously published literature [51,52]. Briefly, 17 mg PLGA, 1 mg Cy5-labeled PLGA, and 2 mg DOTAP were dissolved in 2 mL of acetone and were added dropwise to 20 mL of poloxamer 188 0.5% *w*/*v*. The organic phase was evaporated for 2 h under magnetic agitation (Multi-Stirrer, Magnetic Stirrer Velp^®^ Scientifica, Usmate Velate, Italy). The NPs were then centrifuged at 9,700 RPM for 10 min to remove excess surfactant (ALC multispeed centrifuge PK 121, Camlab, Cambridge, UK) and the pellet was resuspended in MilliQ and stored at 4 °C until further use. Purified NPs were lyophilized (LyoLab 3000, Heto-Holten, ThermoFisher Scientific, Waltham, MA, USA) in pre-weighed Eppendorf tubes, and the weight yield% (WY%) was calculated as follows:

WY% = (mg lyophilized product/mg total materials) × 100(1)

#### 2.2.2. Surface Modification with HA12

Purified NPs were engineered on the surface by adding HA12 via charge interaction. Based on the amount of NPs recovered, the amount of cationic lipid in the formulation was calculated and used to determine the amount of HA12 to be added. In particular, solutions at different HA12 concentrations were prepared, and 50 µL of stock solutions was added to 300 µL of a prepared NP suspension under magnetic stirring. The concentration was calculated to achieve different N:O molar ratios (N = quaternary amines from the DOTAP molecules, O = acid residues on HA12) ranging from 1:0.025 to 1:2. The HA12 solution was added in 5 µL drops over 2 min. The suspension was left under stirring for 1 h and then characterized.

#### 2.2.3. Chemico-Physical Characterization

The size, Polydispersity Index (PDI), and Z-Potential of the NPs were measured by diluting 10 μL of purified NPs in 1 mL of MilliQ water (final concentration ~0.01 mg/mL) and analyzed using Photon Correlation Spectroscopy (PCS) (Zetasizer Nano ZS, Malvern, Malvern, UK). All samples were analyzed in triplicate at room temperature, and each measurement was made on three different NP formulations.

#### 2.2.4. Morphological Characterization

Morphology of NPs was evaluated using Atomic Force and Electron Microscopy. AFM analysis (AFM, Park Instruments, Sunnyvale, CA, USA) was performed at RT, operating in non-contact mode using triangular silicon tips. The resonant frequencies of the cantilever were found to be about 160 kHz. Before the analysis, NPs were diluted to 0.01 mg/mL in MilliQ water and applied to a small mica disc. Excess water was removed before analysis. The topographical images were flattened using second-order fitting to remove sample tilt.

The structure of the samples was also analyzed by scanning transmission electron microscopy (STEM FEI Nova NanoSEM 450, Bruker, Billerica, MA, USA). Briefly, a drop of the same diluted suspension (0.01 mg/mL) used for AFM imaging was placed on a 200-mesh copper grid (TABB Laboratories Equipment, Berks, UK), allowed to adsorb, and the excess was removed using filter paper. All grids were analyzed using the transmission electron microscope operating at 25 kV using a STEM II detector in Field free mode.

#### 2.2.5. Residual Poloxamer 188

The residual surfactant in the NP matrix was evaluated by an already published colorimetric method [53,54]. About 2 mg of NPs was solubilized in 0.5 mL of DCM and then added to 10 mL of water. After evaporation of DCM, the suspension was filtered through cellulose nitrate filter, porosity 0.45 m (Sartorius, Firenze, Italy), to remove the precipitated PLGA. Then, 2 mL of the aqueous solution was treated with 2 mL of 0.5% *w*/*v* BaCl_2_ in HCl 1 N and 0.5 mL of I_2_/KI (0.05 M/0.15 M) and incubated for 10 min in the dark. Poloxamer 188 concentration was calculated using a spectrophotometer (Model V530, Jasco, Cremella, Italy) measuring the absorbance at 540 nm, using a calibration curve from stock solutions of poloxamer 188 prepared under the same experimental conditions. Linearity was found in a range of 4–48 µg (R^2^ = 0.9927). Due to the sensitivity to heat and light of the aqueous solution of I_2_/KI, the calibration curve was calculated fresh before analysis. The analysis was performed in triplicate on three different NP formulations. The residual poloxamer 188 was calculated as follows:

% RP = (mg poloxamer 188 from quantification/mg NP) × 100(2)

### 2.3. In Vivo Biodistribution

All procedures on mice were conducted at CSSI (Centro Servizi Stabulario Interdipartimentale) and approved by the Ethical Committee of University of Modena and Reggio Emilia (Prot. N. 106 22/11/2012) and by the Italian Ministero della Salute (346/2015-PR). *Rho^P23H^*^/+^ mice [55] were obtained from Dr K. Palczewski and maintained in a 12 h light/dark cycle with free access to food and water. NPs were intravitreally injected in *Rho^P23H^*^/+^ mice at the age of 14 days after birth (PN14). Mice were anesthetized with an intraperitoneal injection of avertin at a dose of 250 mg/kg body weight, a 34 GA needle was inserted adjacent to the limbal border of the cornea and 0.5 µL of fluorescently labeled HA12-coated or uncoated NPs was injected into the vitreous at a concentration of 8 mg/mL. Thus, 24 h after administration, animals were sacrificed, eyes were removed, fixed in Davidson’s fixative for 24 h, embedded in paraffin, and sectioned with a microtome [56]. For cell-type identification, immunofluorescence was performed with anti-Rhodopsin clone 1D4 (1:500), anti-Iba1 (1:100) as primary antibodies, and AlexaFluor 488 anti-mouse or anti-rabbit (1:1000) as secondary antibodies. Nuclei were stained with 0.1 µg/mL DAPI. Slides were mounted using Mowiol 4-88 and images were acquired using an SP8 confocal microscope (Leica, Heidelberg, Germany) with a 40X oil objective, equipped with white-light laser.

### 2.4. In Vitro Tests

#### 2.4.1. Cell Viability

Toxicity of NPs was assessed as previously published [57]. Briefly, 661W-A11 cells were cultured in 96-well plates at a density of 6000 cells/well and increasing dilutions of NPs were added to the culture medium. After 48 or 72 h, the medium was aspirated and the cells in each well were incubated for 2 h at 37 °C with 50 µL of 1 mg/mL 3-(4,5-dimethylthiazol-2-yl)-2,5-diphenyl tetrazolium bromide (MTT) diluted in the culture medium. The supernatant was removed and the purple formazan crystals were dissolved in 100 µL of isopropanol and shaken for 10 min. Lastly, the optical density (OD) was measured at 570 nm using a microplate reader (Labsystems Multiskan MCC/340, Fisher Scientific, Rodano, Italy).

#### 2.4.2. Cell Uptake Studies

661W-A11 cells were seeded onto laminin-coated glass coverslips in a 24-well plate at a density of 20,000 cells/well and 8 µg/mL of NPs was added to the culture medium. After incubation for 1 or 24 h, cells were fixed with 2% paraformaldehyde for 10 min. Cell cytoskeleton was labelled by immunofluorescence. Briefly, the cells were blocked and permeabilized with 3% bovine serum albumin and 0.1% Triton X-100, then incubated with the primary antibody anti-α-Tubulin (1:100) for 1 h and secondary antibody AlexaFluor 488 anti-mouse (1:1000) for 1 h and nuclei were stained with 0.1 µg/mL DAPI. Slides were mounted using Mowiol 4-88 and images were acquired using a confocal microscope.

### 2.5. Thermosensitive Hydrogel (TSH)

The formulation of the TSH was optimized by changing the concentration of HA 330 kDa (HA330) and poloxamer 407 (P407). Empty TSH was formulated by dissolving different amounts of HA330 in MilliQ water at 50 °C under magnetic stirring for about 30 s, ranging from 0.5 to 1% *w*/*v*. After complete dissolution, samples were cooled to 4 °C. Then, different amounts of P407 were added from 5 to 15% *w*/*v*, and samples were left under agitation at 4 °C overnight.

To formulate TSH loaded with NPs, the NP suspension was prepared and used instead of water during the formulation of the TSH. HA330 was added to the NP suspension and samples were heated to 50 °C for 30 s under stirring. Samples were immediately cooled to 4 °C, P407 was added, and samples were left under agitation overnight at 4 °C. The impact of NP concentration on the rheological properties of the resulting TSH was also evaluated by preparing hydrogels with NP suspensions ranging from 4 to 12 mg/mL.

### 2.6. Synthetic Vitreous (SV)

To investigate the mobility of the NPs loaded in the TSH without the use of animal models, a model for synthetic vitreous (SV) was optimized and characterized starting from literature protocols [58]. Briefly, HA1100 was dissolved in MilliQ at 60 °C under magnetic stirring. After complete dissolution, Agarose was added while keeping the temperature at 60 °C. Eventually, samples were cooled and stored at 4 °C until further use. Details on the formulated SVs and their characterization are provided in the Results.

### 2.7. Rheological Measurements

Rheological properties of the TSH and the SV samples were evaluated with a Haake Mars Rheometer (ThermoFisher Scientific, Waltham, MA, USA), using a conic geometry (1°) of 35 mm diameter. The gap size was 52 µm and the volume of the sample analyzed was 200 µL. All analyses were performed using the RheoWin Data Manager software (version 4.82.0002, ThermoFisher Scientific, Waltham, MA, USA).

The viscosity of synthetic vitreous (SV) samples was measured by applying a shear rate from 0.01 to 300 s^−1^ at 37 °C, and results were fitted with the Carreau-Yasuda mathematical model to obtain the absolute viscosity (η_0_). An amplitude sweep from 10 to 600% was performed at constant parameters of 1 Hz and 37 °C to obtain the storage modulus G’ of the gels, as a mean of values on the linear-viscoelastic regime (LVR). To measure the gelation temperature (Tgel) of the TSH samples, a 20-step ramp in a range of 5–50 °C at a rate of 0.075 °C/sec was performed at a constant frequency f = 1 Hz and a constant shear stress τ = 1 Pa. G’ and G” were recorded at each step and the gelation temperature was obtained at the crossover.

### 2.8. NP Diffusion through TSH

NP diffusion through the TSH was evaluated by confocal imaging using the optimized SV as a model. The SV was heated to room temperature, then 200 µL was put in the wells of an 8-well glass-bottom plate (Ibidi, Gräfelfing, Germany), covered with Parafilm, and incubated at 37 °C for 1 h. After that, 20 µL of cold (4 °C) Cy5-labeled NP suspension or NP-loaded TSH was injected into the SV. Confocal imaging started right after the injection, keeping the temperature at 37 °C. The laser was set to acquire the signal of the Cy5 (ex. 650 nm; em. 670 nm), and images were acquired every 6 h for a total of 48 h using a 10x objective. Each time point consisted of 35 Z-stack images with a 5 µm Z-step. Two fields were imaged, i.e., the injection site and the opposite corner of the well (5 mm distance). After acquisition, images were processed with Fiji ImageJ: Z-stacks were collapsed to maximum intensity per each time point. A threshold was set to remove background signal, and total fluorescent intensity was measured for each time point and for each field. Each sample was analyzed in triplicate. Fluorescence intensity% was calculated as follows:

FI% = FI in the analyzed field/ (FI at injection site + FI opposite corner) × 100(3)

### 2.9. Statistical Analysis

Statistical analysis was performed using the Mann–Whitney test for pairwise comparisons using GraphPad Prism 6 (GraphPad Holdings, San Diego, CA, USA). Significance is indicated in the figures as * *p* < 0.05. All samples were performed with *n* > 3 and expressed as an average with standard deviation (SD).

## 3. Results

### 3.1. Formulation and Characterization of NPs

Starting from an already published nanoprecipitation protocol, hybrid NPs composed of the biodegradable and biocompatible polymer poly(lactic-co-glycolic) acid (PLGA) and the cationic lipid dioleoyl-trimethylammonium propane (DOTAP) were formulated and characterized (Table 1). Dimensional analysis evidenced a monomodal population with a size ∽240 nm, with low dispersity, demonstrated by a PDI < 0.2. The Z-potential was strongly positive, over +35 mV, as expected due to the presence of the cationic lipid. The weight yield was calculated to be 85 ± 6%. Moreover, the amount of residual poloxamer 188 was calculated and accounted for about 10% of the total weight of the NPs, which is in line with previous studies for similar polymeric and hybrid NPs [59].

**Figure 1:**
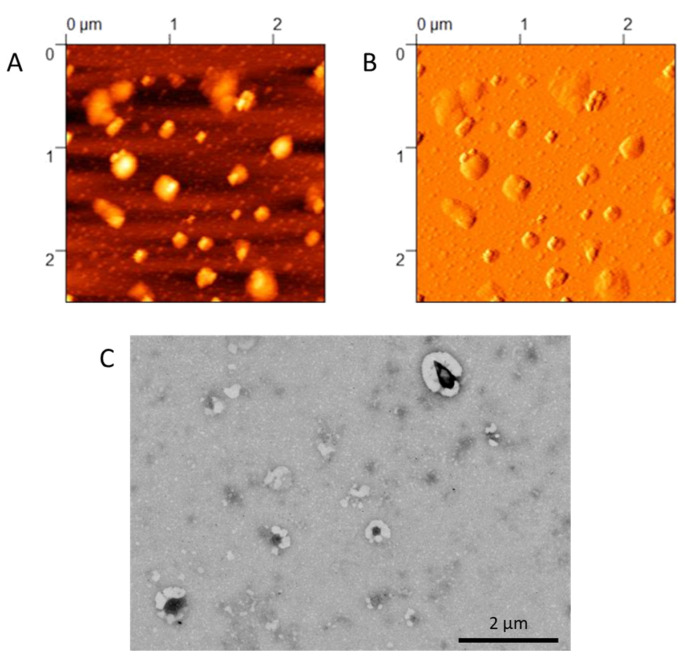
Morphological analysis of HA12-coated NPs, at N:O ratio 1:1. (**A**) AFM “Topography” image, (**B**) AFM “Error Signal” image. (**C**) STEM images.

The formation of the HA12 coating was optimized by evaluating the effects of adding different molar ratios based on the cationic nitrogen of the DOTAP and negative oxygen on the HA12 (N:O ratio) from 1:0.025 to 1:2 (Table 1). Starting from the sample with the lowest amount of HA12, no differences in the Z-potential were noticed with the NPs remaining cationic. This suggested that this amount of HA12 was too low to produce an effective coating. Increasing the amount of HA12 between 1:0.05 and 1:0.5 caused rapid aggregation, which could be ascribed to the incomplete formation of the coating, with the polyanionic molecules extending from one particle to another, causing aggregation between the molecules. Similar effects have been reported in the literature, in which a low concentration of polyanion resulted in the entanglement of the polymeric chains, promoting their aggregation and hampering the colloidal stability [60,61]. A further increase in the N:O ratio over 1:1 resulted in the formation of stable and homogenous NPs with a size around 260 nm and a good homogeneity (PDI < 0.3). Notably, the Z-potential shifted to negative values (∽ −30 mV), suggesting the formation of the HA12 layer on the surface of the cationic NPs. Further addition of HA12 at a 1:2 ratio also resulted in homogeneous and stable NPs, with a size < 300 nm and low PDI, but the ratio of 1:1 was deemed the most promising as it could produce suitable NPs while using the minimum amount of HA12. The presence of this stable coating was also confirmed by Atomic Force (AFM) and Scanning Transmission Electron microscopy (STEM) analysis (Figure 1). AFM images showed the presence of flattened material surrounding the more rigid PLGA-based core, while STEM images revealed the presence of a lighter corona around an electron-dense core, suggesting a distinct deposition of HA12 around the surface of the hybrid NPs.

### 3.2. In Vivo Biodistribution and Microglia Volocalization

**Figure 2:**
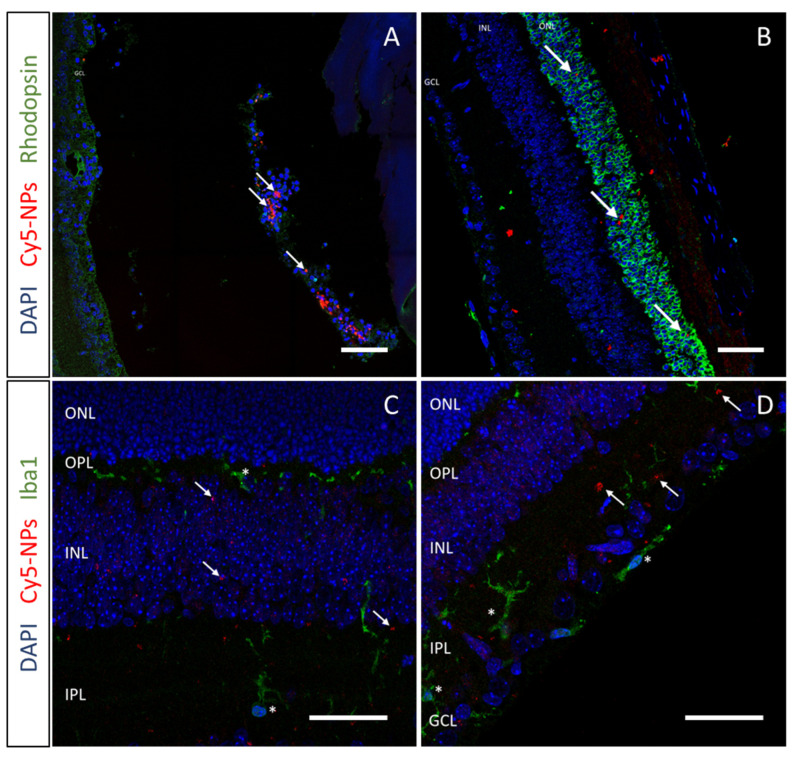
In vivo biodistribution study. Confocal images of sagittal eye sections after intravitreal injection of (**A**) uncoated NPs, (**B**) HA12-coated NPs. Red: Cy5-labeled NPs; Blue: DAPI-labeled nuclei; Green: Rhodopsin labelling photoreceptors. (**C**,**D**) Evaluation of microglia in retinal tissue after injection of HA12-coated NPs. Red: Cy5-labeled NPs; Blue: DAPI-labeled nuclei; Green: Iba-1 labelling microglia cells. Scale bar 40 µm. Arrows indicate NPs, stars indicate Iba-1-positive cells. ONL: Outer nuclear layer; OPL: Outer plexiform layer; INL: Inner nuclear layer; IPL: Inner plexiform layer; GCL: Ganglion cell layer.

To assess the retinal targeting potential of these hybrid NPs, an in vivo biodistribution study was performed. The experiment was performed using a murine line bearing a mutation in the rhodopsin gene (*Rho*) that causes slow-progression retinal degeneration, where photoreceptor cell death starts to be detected at PN15. The choice of a disease model allowed us to define whether the pathological environment may affect NP biodistribution. Mice were intravitreally injected at PN14 with 0.5 µL of a suspension of fluorescently labeled NPs, with or without the HA12 coating, and analyzed one day later. To assess the distribution of NPs in the retinal tissue, photoreceptor and microglia cells were labelled. Confocal imaging evidenced the different behavior of the two formulations: uncoated NPs were unable to disperse through the vitreous due to their cationic surface that strongly interacted with the anionic polymers in the vitreous matrix. These NPs accumulated at the site of injection and were uptaken by cells in the vitreous (Figure 2A). On the other hand, HA12-coated NPs were abundantly found inside the retinal tissue up to the photoreceptor cell layer, indicating that the presence of the coating improved the mobility of NPs through the vitreous towards the retina (Figure 2B).

Microglia are activated in the degenerating retina and may promptly phagocytize the injected NPs. Thus, microglia cells were highlighted to investigate this possibility. Data evidenced no colocalization between NPs and microglia cells, suggesting a low in vivo uptake of these NPs at the concentration used (Figure 2C,D).

### 3.3. In Vitro Uptake Studies

Data collected in vivo suggested that HA12-coated NPs were the most promising NPs to deliver drugs to photoreceptor cells. Hence, HA12-coated NPs were further investigated with toxicity and uptake studies performed in vitro in a photoreceptor cell line, 661W-A11 [49].

**Figure 3:**
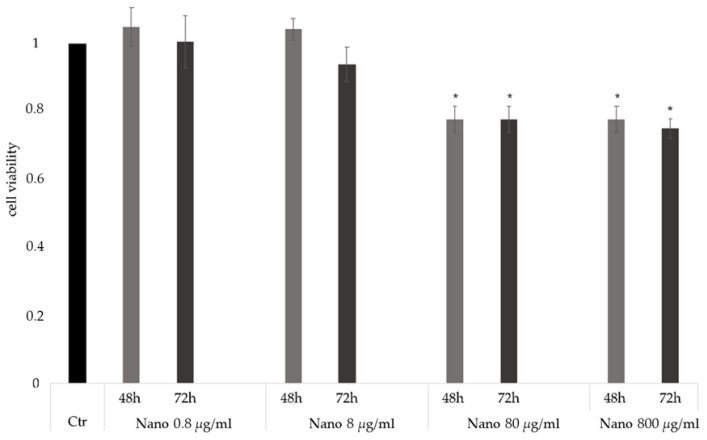
In vitro toxicity study. Cell viability of 661W-A11 cells after administration of HA12-coated NPs diluted in cell medium at 0.8, 8, 80, and 800 µg/mL. Mean and SD, n > 3, with *p* = * < 0.05 calculated using the Mann–Whitney Comparison test.

Toxicity studies were performed to assess the tolerated concentration of coated NPs to be further used in the uptake studies (Figure 3). Reductions in cell viability were not observed with doses up to 8 µg/mL of NPs over 72 h of exposure. Increasing the concentration of NPs to 80 or 800 µg/mL induced a slight toxicity in cells, reducing the cell viability by about 25% with a dose-dependent trend. Thus, 8 µg/mL was chosen as the working dilution for further experiments, as this was the highest concentration that did not significantly affect cell viability.

**Figure 4:**
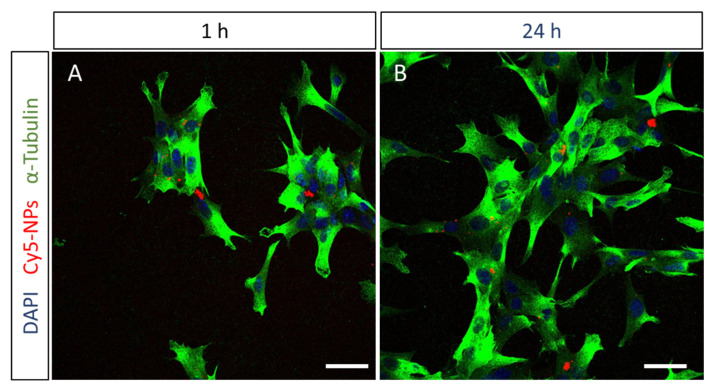
In vitro uptake study. Confocal images of 661W-A11 photoreceptor cells (**A**) 1 h and (**B**) 24 h after administration of HA12-coated NPs. Red: Cy5-labeled NPs; Blue: DAPI labelling nuclei; Green: α-Tubulin labelling the cytoskeleton. Scale bar 40 µm.

To study the ability of photoreceptor cells to uptake the HA12-coated NPs, 661W-A11 cells were co-cultured with fluorescently labeled NPs. Cells were fixed at 1 h and 24 h after administration and NP internalization was assessed by staining the nucleus and the cytoskeleton to distinguish the cytoplasmic and nuclear compartments. Images acquired at 1 h already showed the presence of NPs in the cytoplasm of some cells, which was confirmed after 24 h (Figure 4). Based on these results, together with the in vivo biodistribution study, we propose that these NPs can be a promising tool to deliver drugs to the retina.

### 3.4. Formulation of the TSH

After demonstrating the ability of HA12-coated NPs to reach the retina after IVT injection, the formulation of an NP-loaded thermosensitive hydrogel was optimized. The aim was to have a hydrogel with a liquid-like behavior at cold temperatures, promoting a good injectability and a gelation point around 35 °C, in order to form a hydrogel in vivo. TSH formulations were produced with poloxamer 407 (P407) and hyaluronic acid 330 kDa (HA330). A first screening was performed evaluating the effect of both polymers individually on the gelation temperature (Tgel). First, hydrogels with 15% P407 and HA330 from 0.1 to 1% were tested, showing that the Tgel is independent from the amount of HA330 (Table 2). Thus, 0.5% was chosen for further formulations, to balance the need for biocompatibility together with low viscosity of the formulation. Hydrogels were then formulated with 5–15% P407, evidencing the tight relationship between the concentration of P07 and the Tgel (Table 3): only formulations with 10 and 15% P407 showed a Tgel around 35 °C, being, respectively, around 46 and 25 °C. While it would have been possible to further optimize the empty formulation of TSH to reach Tgel of 35 °C, the influence of NPs in the matrix of a hydrogel is also a crucial parameter to consider, as it can directly affect the Tgel. Therefore, the hydrogel with 10% P407 and 0.5% HA330 was formulated and loaded with either uncoated or HA12-coated NPs to investigate their impact on the gelation.

Different concentrations of NPs were used to assess the loading capacity of the TSHs and their impact on the Tgel. NPs at 4, 8, and 12 mg/mL were produced and characterized (Table 4). Data evidenced that 12 mg/mL NPs were too concentrated to have reproducible formulations: in fact, both coated and uncoated NPs showed the formation of aggregates with higher polydispersity compared to lower concentrations. This also resulted in poor reproducibility when testing the Tgel of the TSH formulations, as suggested by the high SD values of these samples. On the contrary, hydrogels prepared with NP suspensions at 8 and 4 mg/mL showed more reproducible results, all of them having a Tgel in a range 34–40 °C. Specifically, the formulation of TSH produced with uncoated or coated NPs at 8 mg/mL was deemed the most promising to be further tested: the Tgel of these hydrogels was around the desired temperature of 35 °C and, at the same time, NPs were at the highest concentration.

### 3.5. Mobility of NPs through the TSH in Synthetic Vitreous

With the aim of evaluating the behavior of the TSH in a vitreous environment, an in vitro model of the vitreous was developed. The formulation of a model for synthetic vitreous (SV) was optimized by adapting literature protocols, based on hyaluronic acid HA1100 (MW 1100 kDa) and Agarose. Formulations with different amounts of these polymers were tested for viscosity and storage modulus G’ to match with values reported for the human vitreous. In particular, the composition was modified in order to obtain a viscosity around 0.1 Pas and a G’ < 3 Pa, and the optimal composition was found to be 0.25% *w*/*v* HA1100 and 0.05% *w*/*v* agarose (Table 5) [62,63]. This formulation was used to simulate the vitreous in further experiments.

**Figure 5:**
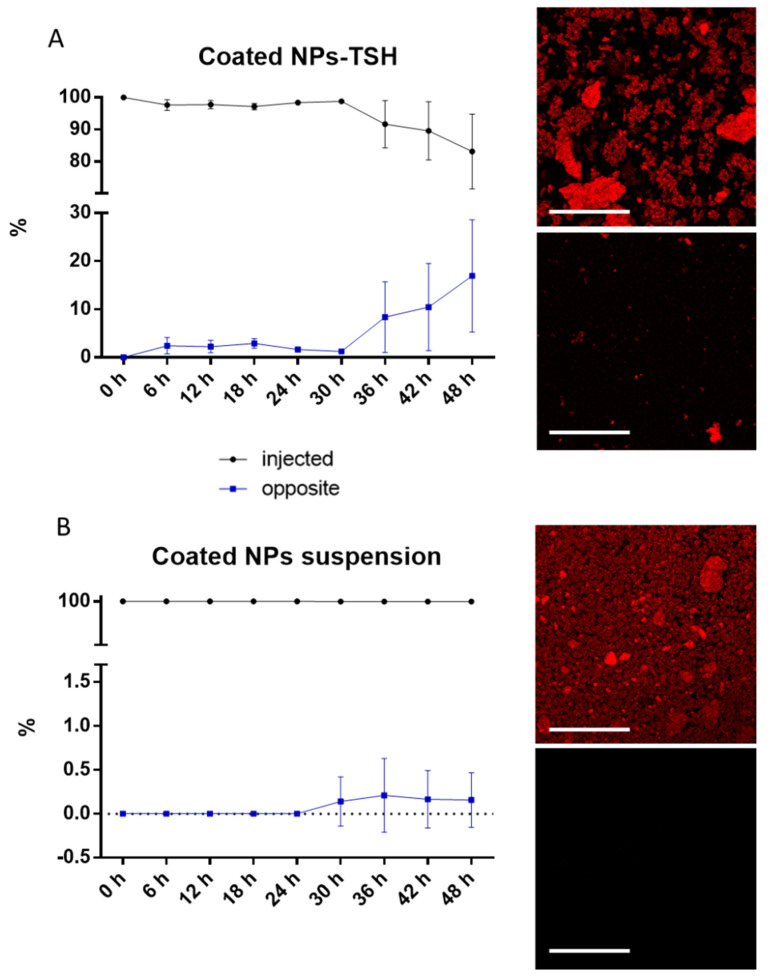
Fluorescence intensity% of NPs after administration in synthetic vitreous with representative confocal images at 48 h. Black line: fluorescence intensity at the injected site. Blue line: fluorescence intensity at the opposite corner. (**A**) Mobility of HA12-coated NPs in the TSH formulation. (**B**) Mobility of HA12-coated NPs in water suspension. Mean and SD, *n* = 3. Scale bar: 100 µm.

The mobility of NPs loaded into TSH was evaluated using the optimized SV as a model. Both the HA12-coated NPs embedded in the TSH formulation and NPs as a suspension were injected in the synthetic vitreous to evaluate their mobility. When NPs were embedded in the TSH, only 7% of NPs were found at the opposite corner of the well after 36 h and increased to around 15% after 48 h incubation (Figure 5A). This 36 h delay suggests that the hydrogel could help control the release of these retinal-targeted NPs, enhancing the therapeutic efficacy. On the other hand, the suspension of coated NPs was expected to be highly mobile when in the SV, similarly to what was observed in vivo. Surprisingly, they rapidly precipitated to the bottom of the dish, and no fluorescence was observed after 48 h at the opposite corner (Figure 5B).

## 4. Discussion

The development of non-invasive delivery strategies is one of the major challenges in the field of treating retinal pathologies. The retina is well protected by the inner limiting membrane, which separates it from the vitreous and the blood–retinal barrier, together limiting diffusion of therapeutics to the retinal cells. Moreover, free drugs injected in the vitreous are often rapidly degraded or eliminated, reducing their therapeutic efficacy. Finally, common administration routes for the retina are often invasive, producing inflammation and reducing the compliance of patients. In this work, we optimized an advanced drug delivery system, composed of coated hybrid nanoparticles embedded in an injectable hydrogel that undergoes gelation in vivo. This system can allow for the slow release of nanoparticles and, thanks to their HA coating, their passage towards the retina can be improved, which could positively affect their therapeutic efficacy and reduce the invasiveness of the approach.

Retinal-targeted hybrid nanoparticles (NPs) have been demonstrated to have great promise when optimized to transport various biological molecules to the retina, as they combine the characteristics of lipidic and polymeric systems for improved stability, enhanced drug loading, lower degradation kinetics, and decreased cell toxicity [64,65,66,67], but they can still require repeat injections, diminishing their overall efficacy and desirability. Here, hybrid NPs were successfully formulated with a single-step nanoprecipitation protocol, followed by surface modification via charge interaction between the core and the HA. The optimized NPs had a diameter of approximately 260 nm, which can be beneficial for an extended half-life and delivery through the vitreous to the retina. The optimized biodegradable NPs, composed of PLGA, Dotap, and hyaluronic acid, hold especially high potential to encapsulate, adsorb, or incorporate a variety of therapeutic molecules. The hydrophobic matrix of PLGA and Dotap will aid in the ability to incorporate small lipophilic molecules [68,69,70], while the cationic charge of Dotap could be advantageous to enhance the loading efficiency of anionic molecules. Finally, the presence of HA enhanced the biocompatibility and mobility of NPs in the vitreous humor, as demonstrated by the fact that uncoated NPs became trapped and did not move through the vitreous, while the HA coating allowed for diffusion towards the retina. This ultimately improved cellular uptake in the retina, most likely due to the interaction with the CD44 receptors, as has previously been suggested in the literature [71,72,73]. This nanosystem represents a highly versatile tool to deliver therapeutics to the retina, as demonstrated by its targeting potential and its ability to diffuse throughout the vitreous and reach the retina, where NPs could be endocytosed by photoreceptor cells, while also representing a system that could be highly compatible with various drug types, if compared to the non-hybrid or non-coated systems alone, as the anionic HA layer on the surface is expected to help entrap cationic molecules [74,75,76].

Notwithstanding the possibility of using HA-coated NPs to deliver therapeutics to the retina, strategies to reduce the number of intravitreal injections needed by prolonging the stability of the NPs were developed. With this aim, HA-coated NPs were embedded into an injectable thermosensitive hydrogel, which was optimized to have a gelation point of around 35 °C. This temperature would allow it to be easily injectable in its liquid form but would form a scaffold when administered in vivo, reaching biological temperatures in the eye. This was achieved by using 10% P407, 0.5% HA330, and 8 mg/mL NPs. These parameters were optimized for IVT-injectable scaffold systems, requiring minimized injection volumes and maximized NP loading to be efficacious. This is because concerns arise that injecting a hydrogel would overly increase interocular pressure and cause damage [77,78]. While this is true, especially in such a small model, such as in a mouse, advanced methodologies for higher-order animals (i.e., rabbits) have already started to overcome this issue by performing a small vitrectomy before gel injection to reduce the overall pressure. This work showed that such a procedure in the rabbit retina did not lead to inflammation or other damage, opening the possibility for more detailed studies going forward [79].

To demonstrate the ability of the TSH to reduce the mobility of NPs in the vitreous, enhancing the timeframe of therapeutic efficacy, NP-loaded TSH and an NP suspension were compared after injection in a synthetic vitreous model. Results evidenced that, contrary to the rapid diffusion of NPs observed in vivo, the THS was able to delay NP mobility for up to 36 h after administration. Surprisingly, NPs in suspension rapidly sedimented at the bottom of the dish and were not able to move in the SV. This demonstrates a missing piece in the use of this in vitro model just for NP assessment, as the phenomenon could be explained by the different microenvironment formed at the interface between the sample and the SV. For the NPs alone, the aqueous phase, even if heated to the solidification temperature of the gel (37 °C), dilutes the gel at the point of injection, leading to a loss of viscosity and allowing for the precipitation of the NPs down onto the surface, where they can no longer move throughout the gel. On the other hand, increasing the viscosity and osmotic pressure of the medium by adding the HA330 and P407 enhanced the colloidal stability and the similarity between the hydrogel and the SV resulting in a reduced osmotic gradient that would favor suspension of the NPs [80,81,82,83]. While these results would closely mimic the results of the in vivo retinal injection of the loaded TSH, the artifact with the NPs alone demonstrated the need for more advanced in vivo studies to precisely assess the impact of the hydrogel on the mobility of NPs.

Recent studies over the last two years have addressed similar approaches based on hydrogels or NPs for ocular applications. The use of hydrogel alone has been investigated by López-Cano et al., demonstrating promising results; however, its efficacy was only tested with small molecules in vitro; therefore, the stability and drug release could differ after in vivo injection, demonstrating rapid degradation or clearance as often seen for non-protected small molecules [84]. On the other hand, Suri et al. studied NPs of PLGA and Chitosan to protect and deliver sirolimus to the retina for the treatment of age-related macular degeneration [85]; however, NPs alone might not ensure a prolonged release of the drug as they are often rapidly cleared from the eye, requiring multiple injections. Thus, the combination of NPs and hydrogels seems to be the most effective strategy going forward to achieve both prolonged release and protection of the drug. To this end, Hsu et al. developed a hydrogel based on hyaluronic acid loaded with PLGA NPs, using bovine serum albumin as a model drug. While showing promising results in terms of stability and drug release, the hydrogel required chemical cross-linking before administration. This led to a more stable system, but highly viscous cross-linked hydrogels cannot be injected and must be implanted, leading to a more invasive approach [86]. Finally, injectable thermosensitive hydrogels loaded with chitosan-based NPs were investigated by Taheri et al. This system required the addition of the semi-synthetic cellulose derivative HPMC to reach the desired gelation point and higher concentrations of poloxamer 407 compared to our system [87].

Altogether, our thermosensitive NP-loaded hydrogel was optimized to be versatile to overcome many of these barriers: (1) hybrid NPs to load and protect a variety of molecules; (2) HA coating on NPs to improve mobility through the vitreous towards the retina; (3) thermosensitive hydrogel for sustained release; (4) gelation at physiological temperature allowing for injectability with gel formation upon administration. This work is a first step in creating a more patient-friendly and new curative option for retinal diseases.

## Figures and Tables

**Table 1:** Chemico-physical characteristics of NPs.

N:O Ratio	Size (nm)	PDI	Z Potential (mV)
1:0 ^†^	239 ± 18	0.16 ± 0.04	+39 ± 2
1:0.025	306 ± 12	0.29 ± 0.05	+38 ± 7
1:0.05	>1000	/	/
1:0.1	>1000	/	/
1:0.2	>1000	/	/
1:0.5	846 ± 147	0.78 ± 0.11	−30 ± 7
1:1	257 ± 6	0.21 ± 0.04	−35 ± 5
1:2	298 ± 16	0.25 ± 0.07	−38 ± 6

^†^ 1:0 ratio refers to uncoated NPs.

**Table 2:** Effect of the HA330 concentration on the Tgel of different empty TSH formulations with 15% P407.

HA330	Tgel °C (SD)
0.1%	27.2 (2.0)
0.25%	24.2 (0.3)
0.5%	25.7 (3.0)
0.75%	23.1 (0.1)
1%	26.6 (1.9)

**Table 3:** Effect of the P407 concentration on the Tgel of different empty TSH formulations with 0.5% HA330.

P407	Tgel °C (SD)
5%	>50
10%	46.2 (3.1)
15%	25.7 (3.0)

**Table 4:** Chemico-physical characteristics of NPs at different concentrations, and effect of the concentration of NPs on the Tgel of a TSH formulation with 10% P407 and 0.5% HA330.

[NPs]	Uncoated NPs			HA12-coated NPs		
	Size nm (SD)	PDI (SD)	Tgel °C (SD)	Size nm (SD)	PDI (SD)	Tgel °C (SD)
4 mg/mL	228 (5)	0.10 (0.05)	40.4 (2.4)	224 (13)	0.16 (0.02)	36.6 (0.9)
8 mg/mL	239 (18)	0.16 (0.04)	34.3 (0.5)	257 (6)	0.21 (0.04)	35.1 (1.1)
12 mg/mL	460 (114)	0.25 (0.18)	39.2 (10.3)	361 (24)	0.30 (0.05)	38.8 (9.4)

**Table 5:** Composition and rheological characteristics of synthetic vitreous models.

Agarose	HA1100	η (Pas)	G’ (Pa)
0.5% *w*/*v*	0.5% *w*/*v*	11.0 ± 1.9	15.1 ± 3.2
0.1% *w*/*v*	0.5% *w*/*v*	5.1 ± 0.5	13.9 ± 2.3
0.1% *w*/*v*	0.25% *w*/*v*	0.5 ± 0.1	0.7 ± 0.1
0.05% *w*/*v*	0.5% *w*/*v*	0.7 ± 0.1	1.2 ± 0.2
0.05% *w*/*v*	0.25% *w*/*v*	0.1 ± 0.0	0.3 ± 0.1

## Data Availability

Raw data are available upon request to the corresponding author.
